# Mechanisms Underlying Stage-1 TRPL Channel Translocation in *Drosophila* Photoreceptors

**DOI:** 10.1371/journal.pone.0031622

**Published:** 2012-02-20

**Authors:** Minh-Ha Lieu, Maximiliano J. Vallejos, Emily Michael, Susan Tsunoda

**Affiliations:** 1 Department of Biomedical Sciences, Colorado State University, Fort Collins, Colorado, United States of America; 2 Department of Biology, Boston University, Boston, Massachusetts, United States of America; Yale School of Medicine, United States of America

## Abstract

**Background:**

TRP channels function as key mediators of sensory transduction and other cellular signaling pathways. In *Drosophila*, TRP and TRPL are the light-activated channels in photoreceptors. While TRP is statically localized in the signaling compartment of the cell (the rhabdomere), TRPL localization is regulated by light. TRPL channels translocate out of the rhabdomere in two distinct stages, returning to the rhabdomere with dark-incubation. Translocation of TRPL channels regulates their availability, and thereby the gain of the signal. Little, however, is known about the mechanisms underlying this trafficking of TRPL channels.

**Methodology/Principal Findings:**

We first examine the involvement of *de novo* protein synthesis in TRPL translocation. We feed flies cycloheximide, verify inhibition of protein synthesis, and test for TRPL translocation in photoreceptors. We find that protein synthesis is not involved in either stage of TRPL translocation out of the rhabdomere, but that re-localization to the rhabdomere from stage-1, but not stage-2, depends on protein synthesis. We also characterize an *ex vivo* eye preparation that is amenable to biochemical and genetic manipulation. We use this preparation to examine mechanisms of stage-1 TRPL translocation. We find that stage-1 translocation is: induced with ATP depletion, unaltered with perturbation of the actin cytoskeleton or inhibition of endocytosis, and slowed with increased membrane sterol content.

**Conclusions/Significance:**

Our results indicate that translocation of TRPL out of the rhabdomere is likely due to protein transport, and not degradation/re-synthesis. Re-localization from each stage to the rhabdomere likely involves different strategies. Since TRPL channels can translocate to stage-1 in the absence of ATP, with no major requirement of the cytoskeleton, we suggest that stage-1 translocation involves simple diffusion through the apical membrane, which may be regulated by release of a light-dependent anchor in the rhabdomere.

## Introduction

Transient Receptor Potential (TRP) channels constitute a superfamily of cationic channels expressed in a diverse array of cell types and systems. Many TRP channels function as key mediators of a variety of sensory transduction pathways, including pain, thermosensation, taste transduction, mechanosensation, and vision [1], [2]. The first TRP channel described, along with its subsequently identified homolog, TRP-like (TRPL), function as the primary light-activated channels in *Drosophila* phototransduction [3]–[6]. TRP channels are statically anchored in the rhabomere, a microvillar-rich compartment specialized for phototransduction. In contrast, the localization of TRPL channels has been shown to be dynamic, and regulated by light [7]–[9]. Translocation of TRPL channels out of the rhabdomere is thought to make them unavailable for signaling, thereby regulating the gain of the light response and contributing to mechanisms of light-adaptation [7], [10].

This signal-induced translocation of TRPL channels is a good model for other TRP channels, and other ion channels, which undergo subcellular trafficking as a means of regulating channel availability. Several studies have provided evidence for the stimulation-induced translocation of intracellular TRP channels to the plasma membrane. For example, TRPV2, found mainly in intracellular pools, translocates to the plasma membrane after stimulation by insulin-like growth factor [11]. Translocation of a complex consisting of RhoA, IP3R, and TRPC1 to the plasma membrane is thought to occur after stimulation by thrombin [12]. Similarly, epidermal growth factor stimulation induces incorporation of mammalian TRPC5 into the plasma membrane [13].

In *Drosophila* photoreceptors, TRPL channels are localized to the rhabdomere in the dark, and with illumination, they translocate to the cell body [7]–[9]. Activation of the major light receptor rhodopsin-1, the effector phospholipase-C (PLC), and the other light activated channel TRP were all found to be required for TRPL channel translocating to the cell body [7]–[9]. With more detailed analyses, we found that TRPL translocation occurred in two distinct stages [8]. TRPL channels first translocate to the neighboring apical/stalk membrane (stage-1), and with longer illumination, translocate to the basolateral membrane of the cell body (stage-2). These stages were also genetically separable since stage-1 was independent of TRP, and stage-2 required the activation of the entire phototransduction cascade and even an eye-specific protein kinase-C (eye-PKC) [8]. Constitutive activation of TRP channels was sufficient to trigger stage-2 translocation of TRPL [8], and consistently, translocation to the cell body was also shown to be dependent on extracellular Ca^2+^ [9].

Much more headway has been made into understanding mechanisms underlying the light-dependent translocations of the G-protein and arrestin proteins in vertebrate and *Drosophila* photoreceptors [14]–[19] . TRPL channels, however, are transmembrane proteins and have longer time-courses of translocation, suggesting different cellular strategies. For example, with hours (versus minutes) required for TRPL translocation to the second stage, it is important to determine whether this change in localization might be due instead to protein degradation/re-synthesis. We investigate the involvement of protein synthesis, and whether active and/or passive transport mechanisms are likely to contribute to TRPL channel translocation, especially for stage-1. We provide evidence that the actin cytoskeleton does not play a major role in the first stage of TRPL translocation. We also show that TRPL translocation to stage-1 is independent of ATP, suggesting that simple diffusion may account for the rapid redistribution of TRPL channels from the rhabdomere to the apical/stalk membrane. Consistent with this hypothesis, we find that increasing membrane sterol composition slows the rate of stage-1 TRPL translocation.

## Results

### Determining if TRPL Channel Translocation Requires New Protein Synthesis

**Figure 1:**
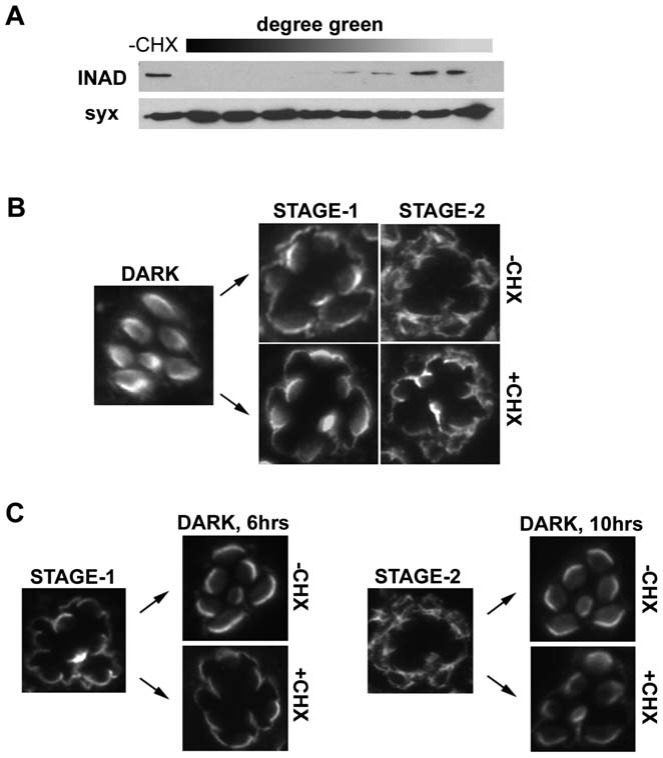
Testing the Requirement of Protein Synthesis for TRPL Channel Translocation. (A) *hs-inaD* transgenic flies (in an *inaD^1^* null background) express INAD protein only when heat-shocked at 37°C. One hour of heat-shock induces INAD expression that can be easily detected by immunoblot analysis (see -CHX). Flies fed green-colored cycloheximide (CHX) for 30 minutes were selected with for abdomens with various degrees of greenness (degree green), heat-shocked, and analyzed by immunoblot analysis (three heads per lane). Degree greenness had an inverse relationship with inducible expression of INAD, indicating that CHX treatment did indeed block protein synthesis. Anti-syntaxin was used as a loading control. (B) Dark-raised wild-type flies were fed +/− CHX (colored green) in the dark for at least 30 minutes, then light-exposed for either 30 minutes (Stage-1) or 12 hours (Stage-2), while remaining on the +/− CHX food throughout the light-exposure. Only flies with dark-green abdomens were selected for sectioning. Shown are representative retinal sections immunostained for TRPL. TRPL channels translocated to both stage-1 and -2 with CHX treatment. Multiple tissue sections were taken from 11 eyes from 8 flies (Dark), 8 eyes from 7 flies (Stage 1, -CHX), 9 eyes from 7 flies (Stage 1, +CHX). (C) Left, Wild-type flies were fed +/− CHX in the dark for at least 30 minutes to inhibit protein synthesis, light-exposed for 30 minutes to induce stage-1 translocation, followed by 6 hours of dark incubation. Only flies with dark-green abdomens were selected for sectioning and immunostaining. TRPL channels relocalized to the rhabdomeres in –CHX flies, but were unable to translocate back to the rhabdomere in +CHX fed flies. Right, Wild-type flies were light-exposed for 12 hours to induce stage-2 translocation, then transferred to +/− CHX food in continuous light for 1 additional hour, followed by dark incubation for 10 hours. Only flies with dark-green abdomens were selected for sectioning and immunostaining. Representative sections show that TRPL channels relocalized to the rhabdomere from stage-2 in both +/− CHX. Multiple sections were taken from 13 eyes from 9 flies (Stage 1), 7 eyes from 5 flies (Dark 6 hrs, -CHX), 12 eyes from 9 flies (Dark 6 hrs, +CHX), 13 eyes from 11 flies (Stage 2), 11 eyes from 9 flies (Dark 10 hrs, -CHX), 16 eyes from 10 flies (Dark 10 hrs, +CHX).

TRPL channels undergo a progressive light-dependent change in localization from the rhabdomere, in the dark, to the neighboring apical membrane, within five minutes and up to four hours of light-exposure (stage-1), and finally, to the basolateral membrane after light-exposures over six hours (stage-2) [8]. Re-localization to the rhabdomere requires dark-incubation of 6 hours from stage-1 and 10 hours from stage-2. No reports, however, have determined whether protein synthesis is involved in either stage of this light-induced translocation, or the re-localization of TRPL channels back to the rhabdomere with dark-incubation. To address this question, we used an assay in which protein synthesis is blocked in living flies. In this protocol, we feed flies the protein synthesis inhibitor, cycloheximide (CHX), mixed with green food-coloring. To determine if this method indeed blocks protein synthesis, we tested the assay with transgenic flies that express an *inaD* under the control of a heat-shock promoter (now referred to as *hs-inaD* flies*)*. Without heat-shock, *hs-inaD* flies do not express INAD protein, while one hour of heat-shock at 37°C induces new INAD protein synthesis detectable by immunoblot analysis (Figure 1A). *hs-inaD* flies fed CHX for at least 30 minutes, and selected for a medium to dark green abdomen, showed no heat-induced synthesis of INAD protein (Figure 1A). Inhibition of protein synthesis was maintained for up to 24 hours (data not shown).

To test if either stage of light-induced TRPL channel translocation from the rhabdomere is dependent on protein synthesis, wild-type flies were fed CHX in the dark for at least 30 minutes, then light-exposed for either 30 minutes or 12 hours, remaining on the CHX food throughout the experiment. We found that even with CHX treatment, TRPL channels displayed normal stage-1 and stage-2 translocation from the rhabdomere to the apical and basolateral membranes, respectively (Figure 1B). To determine if TRPL channel recovery to the rhabdomere from stage-1 is dependent on protein synthesis, dark-raised wild-type flies were fed CHX in the dark for at least 30 minutes to induce protein inhibition, followed by light-exposure for 30 minutes to promote stage-1 of translocation, and subsequent dark incubation for 6 hours, remaining on the CHX food for the duration of the experiment. Interestingly, we found that TRPL channels were unable to translocate back to the rhabdomere from stage-1, remaining in the apical stalk membrane (Figure 1C, left). These results suggest that re-localization from stage-1 is dependent on protein synthesis. Similar experiments were performed for examining relocalization from stage-2. Although redistribution back to the rhabdomeres from stage-2 required at least 10 hours of dark incubation, inhibition of protein synthesis had no effect on this process (Figure 1C). To test whether inhibition of protein synthesis by CHX was less effective during this lengthy experiment (∼22 total hours), we also shortened the amount of time the flies were fed CHX to 11 hours. Wild-type flies were light-exposed for 12 hours to induce stage-2 translocation, and then transferred to CHX food for 1 hour, followed by 10 hours of dark incubation, while remaining on the CHX medium. TRPL channels still underwent normal translocation back to the rhabdomere from stage-2 (Figure 1C, right).

Altogether, our results suggest that both stages of light-induced TRPL channel translocation are independent of protein synthesis, and not likely to be due to a protein degradation/re-synthesis mechanism. TRPL channel translocation back to the rhabdomere from stage-2 also appears to be independent of protein synthesis. In contrast, TRPL channel recovery to the rhabdomere from stage-1 is dependent on protein synthesis, indicating that re-localization from stage-2 occurs via a completely different pathway from re-localization mechanisms from stage-1.

### Stage-1 TRPL Translocation is Not Regulated by *shibire*-Mediated Endocytosis

**Figure 2:**
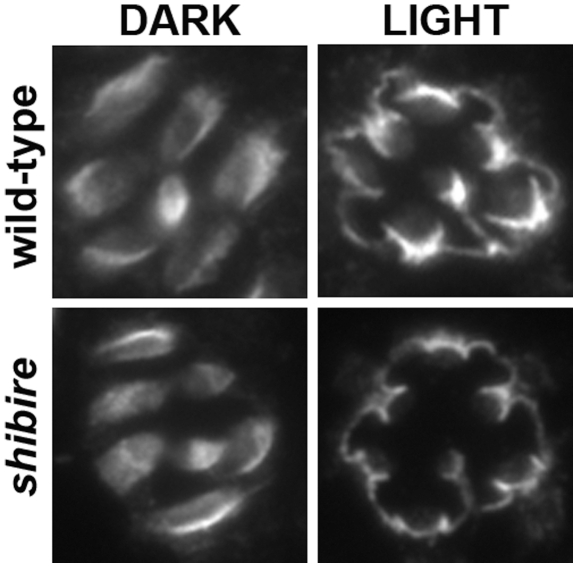
Stage-1 TRPL Translocation is Not Regulated by *shibire*-Mediated Endocytosis. Shown are representative retinal sections of single ommatidia from wild-type and *shibire^ts1^* mutants. Dark-raised flies were first incubated at 30°C to block endocytosis in *shi^ts1^*, indicated by paralysis. Flies continued to be incubated at 30°C but either remained in the dark or were light-exposed for 2 hours. Tissue sections were immunostained for TRPL. TRPL displayed normal light-induced translocation from the rhabdomere to the neighboring stalk membrane. Multiple tissue sections were taken from 14 eyes from nine flies (wild-type, Dark), 10 eyes from 8 flies (wild-type, Light), 10 eyes from 7 flies (*shibire^ts1^*, Dark), 15 eyes from 10 flies (*shibire^ts1^*, Light).

What are the molecular mechanisms underlying the light-induced translocation of TRPL channels? One possibility is that TRPL channels may be incorporated into vesicles at the base of the rhabdomere, similar to rhodopsin-Arr-2 complexes that accumulate in *norpA* and *rdgC* mutants [20]–[22], and transported to downstream subcellular sites. We examined the role of endocytosis using the temperature-sensitive mutant, *shibire* (*shi^ts1^*). *shibire* encodes the GTPase, dynamin, required for “pinching off” of vesicles during endocytosis [23]. At 25°C, *shi^ts1^*mutants are indistinguishable from wild-type, but when the temperature is raised to 29–30°C, *shi^ts1^* mutants display rapid paralysis as a result of disruption in endocytosis [23], [24]. Involvement of *shibire*-mediated endocytosis was previously investigated for TRPL translocation [9], however not specifically for stage-1 translocation. Thus, we examined the immunolocalization of TRPL channels in dark-raised and 30-minute light-exposed *shi^ts1^* mutants incubated at the restrictive temperature. To verify that endocytosis was blocked in *shi^ts1^* mutants, we tested them first for paralysis at the restrictive temperature before using them for immunolocalization studies. We found that TRPL channels were localized to the rhabdomere in dark-raised *shi^ts1^* mutants, and light-exposure resulted in translocation to the stalk membrane, similar to wild-type at 30°C (Figure 2). These results indicate that stage-1 TRPL translocation is independent of *shibire*-mediated endocytosis. For stage-2, the lengthy incubation at the restrictive temperature, unfortunately, resulted in severe retinal degeneration and lethality, making the role of endocytosis in stage-2 translocation inconclusive.

### Characterization of an *Ex Vivo* Retina Preparation

**Figure 3:**
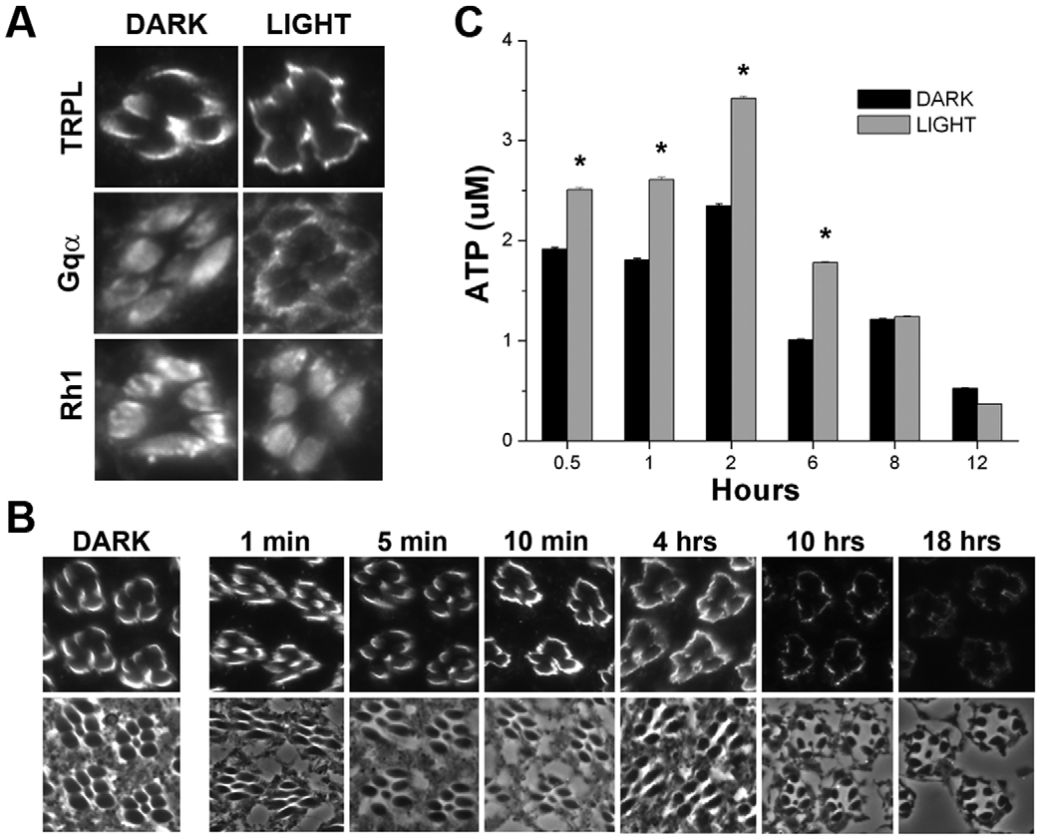
Bisected Head Illumination (BHI) Preparation for Examining Stage-1 TRPL Translocation. (A) Dark-raised wild-type fly heads were removed and bisected under dim red light, then placed in wells of a tissue culture plate containing a buffered bath solution. The plates containing the eyes were light-exposed for 30 minutes or remained in the dark. Eyes were then fixed, sectioned and immunostained for the indicated phototransduction proteins. Note that TRPL and G_q_α displayed normal light-induced translocation, while Rh1 displayed static rhabdomeric localization, as expected. (B) Shown are wild-type retinal sections from eyes in the BHI preparation exposed to light of increasing duration, as indicated. The top panels show TRPL immunostaining and the bottom panels show the corresponding phase-contrast image. Dark-incubated eyes show rhabdomeric TRPL localization. After 10 minutes of light-exposure, TRPL translocates to the stalk membrane, signifying stage-1 translocation. With additional light-exposure, TRPL was never observed to translocate to stage-2, even after 18 hours of light-exposure. TRPL signal was dim after this lengthy 18-hour light-exposure and the photoreceptors appeared slightly degenerated from 10 to 18 hours. Multiple retinal sections were taken from 12 eyes from 9 eyes (Dark), 10 eyes from 5 flies (1 min), 8 eyes from 5 flies (5 min), 7 eyes from 4 flies (10 min), 12 eyes from 7 flies (4 hours), 5 eyes from 4 flies (10 hours), 5 eyes from 3 flies (18 hours). (C) ATP content of eyes at various times incubated in the BHI preparation was quantitated. For each time point, 12 eyes were incubated at room temperature for the indicated times in the dark (black bars; DARK), or dark followed by 30 minutes of light-exposure (grey bars; LIGHT). ATP was quantified from 6 eyes homogenized for each condition; means ± SD from 5 independent experiments are shown. A significant difference (p<0.05, [*]; students t-test) between dark and light conditions was observed from time points from 30 minutes to 6 hours.

To further investigate the molecular mechanisms involved in TRPL channel translocation, we characterized an *ex vivo* preparation that would allow us to apply chemical inhibitors to photoreceptors and then assay for effects on TRPL translocation; we call this preparation the bisected head illumination (BHI) preparation. Fly heads are bisected under dim red light and placed in culture wells containing a bath solution, to which biochemical inhibitors can be added and eyes can be light-exposed or dark-incubated. After treatment, eyes are fixed, sectioned, and immunostained. With bath solution alone, TRPL channels are localized to the rhabdomeres of eyes incubated in the dark (Figure 3A,C). Light-exposure (30 minutes) of eyes in the BHI preparation induced translocation of TRPL channels to the neighboring stalk membrane (stage-1; Figure 3A), similar to what is observed in photoreceptors of light-exposed flies (Figure 1B). As a control, we also examined the visual G_q_α and the major rhodopsin (Rh1). Previous *in vivo* studies have shown that G_q_α, like TRPL, undergoes light-induced translocation to the cell bodies of photoreceptors [14], [25], while Rh1 remains rhabdomeric regardless of light condition [8], [14]. Indeed, in the BHI preparation, G_q_α was rhabdomeric when incubated in the dark, and redistributed to the cell body with light-exposure, while Rh1 was localized to the rhabdomere in both dark and light conditions (Figure 3A). These results suggested that photoreceptors in the BHI preparation were viable and displayed light-dependent localization and translocation of phototransduction proteins similar to studies in which live flies were light/dark-treated.

We examined TRPL translocation in the BHI preparation in more detail. Previously, we found that TRPL channels translocate out of the rhabdomeres to stage-1 (neighboring apical membrane) within 5 minutes of light-exposure and remain in stage-1 for about four hours, then translocate to stage-2 (basolateral membrane) after 6–10 hours of light-exposure [8]. In the BHI preparation, we found that TRPL channels translocate a little more slowly, requiring 10 minutes of light-exposure to reach stage-1 (Figure 3B). With longer light-exposures, however, even up to 18 hours, TRPL channels remained restricted to the apical membrane, and were never observed to translocate to the basolateral membrane (Figure 3B). One possibility is that viability of photoreceptors in the BHI preparation is increasingly compromised over time. Photoreceptor cells indeed appeared progressively more degenerated, especially after 10 and 12 hours of light-exposure (Figure 3B).

Since live cells synthesize ATP as a means for providing cellular energy, we measured ATP levels as a gauge for cell viability. Dark-raised wild-type eyes were incubated in the bath solution for times ranging from 30 minutes to 12 hours, and either remained in the dark or were subsequently light-exposed for 30 minutes. Using a luciferase-based reporter assay on homogenized eyes, we found that levels of ATP progressively decreased, with a time-course roughly corresponding to the photoreceptor cell degeneration observed (Figure 3B). Because absolute ATP levels varied quite drastically from experiment to experiment, control and experimental samples were always quantified simultaneously and averages were taken across multiple independent experiments. At 12 hours, ATP concentration was decreased by more than 50% compared to 30 minutes after head bisection (Figure 3C). One possibility is that TRPL channels are not able to translocate to stage-2 in the BHI assay due to insufficient ATP. We also noticed that at all time points up to 6 hours after head bisection, ATP levels were significantly increased with light-exposure (Figure 3C). This light-induced rise in ATP is also a likely indicator of photoreceptor viability. Thus, we have developed an ex vivo preparation of fly eyes in which the first stage of light-induced TRPL translocation can be studied on a time-course similar to that observed from living flies. Unfortunately, photoreceptor degeneration was observed at the longer incubation times required to examine the second stage of TRPL translocation. For subsequent studies described here, we therefore focus on mechanisms underlying stage-1 TRPL translocation.

### Perturbation of the Actin Cytoskeleton Does Not Affect Stage-1 TRPL Translocation

**Figure 4:**
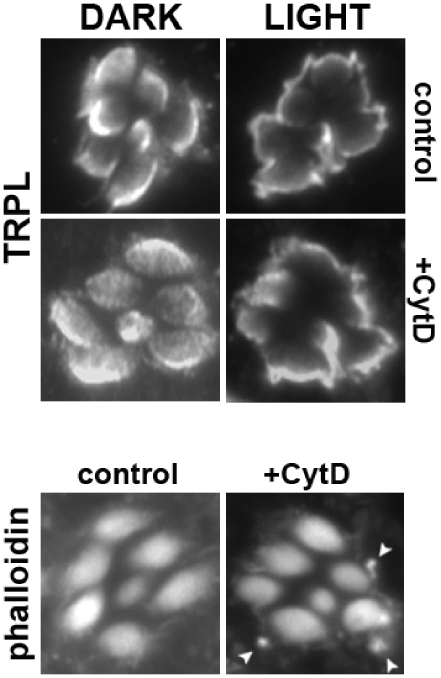
Perturbation of the Actin Cytoskeleton by Cytochalasin-D Does Not Affect Stage-1 TRPL Translocation. Shown are representative retinal sections from dark-raised wild-type eyes from the BHI preparation, incubated in a control bath solution containing 1% DMSO (control) or treated bath solution containing 10 µg/ml of cytochalasin D in 1% DMSO (+CytD). Eyes were incubated in these solutions, in the dark for 1 hour and then either remained in the dark (DARK), or were light-exposed for 10 minutes (LIGHT). Eyes were fixed, sectioned and double-labeled with TRPL and FITC-conjugated phalloidin, which binds F-actin. We observed bright phalloidin-labeled actin-aggregates (arrowheads) near the base of +CytD rhabdomeres that were not present in control eyes. TRPL channels were still able to undergo light-dependent translocation to stage-1 in both conditions. Multiple retinal sections were taken from 9 eyes from 5 flies (Dark control), 8 eyes from 5 flies (Light control), 10 eyes from 6 flies (Dark, +CytD), 6 eyes from 4 flies (Light, +CytD).

Subcellular transport of proteins can occur via active and/or passive mechanisms. Active transport in *Drosophila* photoreceptors would likely involve the major cytoskeletal element, actin, which composes the microvilli of the rhabdomeres. We therefore set out to disrupt the actin cytoskeleton and examine whether TRPL translocation would be affected. Assembly and maintenance of the actin cytoskeleton is regulated by continuous cycles of actin polymerization and depolymerization. We used cytochalasin D (CytD), a membrane permeable mycotoxin known to inhibit actin polymerization [26], [27]. Using the BHI preparation, we were able to treat photoreceptors with CytD. Although phalloidin staining can be used to monitor the actin cytoskeleton, especially after drug treatment, severe disruption has been difficult to attain in insect retinas due to the dense packing of microvilli that make up rhabdoms and rhabdomeres of photoreceptor cells [28], [29]. With this in mind, we increased CytD and DMSO concentrations as much as possible while avoiding significant cell degeneration. We found that one hour CytD treatment at 10 µg/ml in 1% DMSO resulted in bright phalloidin-labeled actin aggregates directly at the base of the rhabdomeres, which were not present in control retinas (Figure 4); similar results were seen with up to 20 µg/ml cytD in 4% DMSO (data not shown). Previous studies in both honeybee retinas and cultured vertebrate cells have demonstrated that phalloidin-staining following CytD treatment results in the appearance of these punctate, actin aggregates or “asters”, composed of densely packed, short actin filaments [28], [29]. We tested for the light-dependent translocation of TRPL with CytD treatment, and observed normal redistribution of TRPL from the rhabdomere to the apical plasma membrane (Figure 4). These results suggest that the actin cytoskeleton is not likely to play a major role in stage-1 TRPL translocation.

### TRPL Channel Localization in the Dark is ATP-Dependent, and Stage-1 Translocation is ATP-Independent

**Figure 5:**
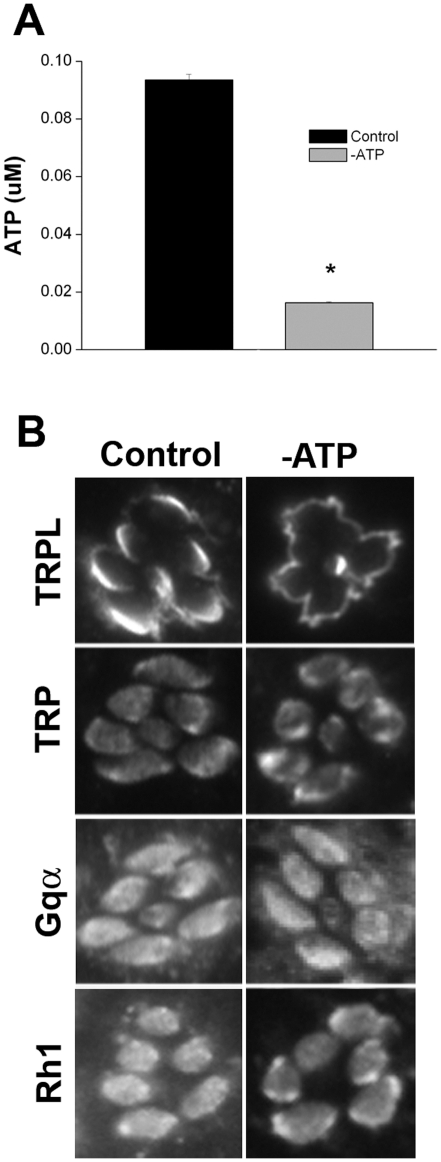
TRPL Channels Translocate to Stage-1 in the Dark with ATP-Depletion. (A) Eyes were incubated at room temperature in the dark for 1 hour in control bath solution (Control), or bath solution supplemented with 2 mM deoxyglucose and 5 mM KCN to deplete ATP (-ATP). To determine ATP levels, 6 eyes from each condition were homogenized, and ATP in the extract was measured using a Luciferase-based reporter assay. Untreated eyes contained 0.09 µM ATP, while eyes treated with deoxyglucose and KCN contained 0.01 µM ATP (Student's t-test, p<0.05). Means ± SD shown are from 3 independent experiments. (B) Shown are representative retinal sections of eyes in control and ATP-depleted conditions described in (A), immunostained for TRPL, TRP, Gqα, and Rh1. Note that all experiments were conducted in the dark. In ATP-depleted eyes, TRPL is mislocalized to the apical plasma membrane, identical to stage-1 localization, while other phototransduction proteins remained rhabdomeric, as expected in the dark. Multiple retinal sections were taken from 12 eyes from 12 flies (control: TRPL and Rh1 double-labeled), 12 eyes from 12 flies (-ATP: TRPL and Rh1 double-labeled), 6 eyes from 6 flies (control: TRP and Gα), 6 eyes from 6 flies (-ATP: TRP and Gα).

To determine whether the light-regulated redistribution of TRPL channels requires energy, we planned to use the BHI preparation to deplete ATP from photoreceptor cells, and then examine whether TRPL channels would translocate to stage-1. To deplete ATP, wild-type eyes were incubated in a glucose-free bath solution supplemented with 2-D-deoxyglucose (DOG) and potassium cyanide (KCN). DOG, a glucose analogue, prevents glycolysis [30], while KCN inhibits mitochondrial cytochrome oxidase, thereby blocking oxidative phosphorylation [31], [32]. Together, these inhibitors have been used to deplete ATP in other cells [33]. First, wild-type eyes were incubated in the dark in either the control bath solution, or bath solution supplemented with DOG and KCN, for one hour. Eyes were homogenized and ATP was quantified using a luciferase-based reporter assay. Indeed, ATP was significantly depleted from retinas with DOG and KCN treatment (Figure 5A).

Next, we performed immunolocalization studies for TRPL in control and ATP-depleted retinas. We found that in ATP depleted conditions, TRPL channels were already localized throughout the apical plasma membrane, even without light-exposure (Figure 5B). In fact, the distribution of TRPL was identical to its localization after stage-1 translocation induced by light, indicating that ATP depletion alone had triggered translocation. Other phototransduction proteins, including the other light-activated channel TRP, G_q_α, and Rh1, displayed normal rhabdomeric localization with ATP depletion (Figure 5B). These results were not so surprising since ATP depletion has been shown to activate TRP channels [34], [35], and indeed, constitutively activated TRP channels (*Trp^P365^*) have been shown to induce TRPL translocation [8]. Thus, it is likely that Ca^2+^ influx through activated TRP channels drives TRPL channel translocation. One possibility is that Ca^2+^ somehow releases an anchor that retains TRPL channels in the rhabdomere.

### Increasing Membrane Sterol Composition Slows the Rate of TRPL Translocation

Our studies thus far suggested that mobilization of TRPL channels to stage-1 was independent of *shibire*-mediated endocytosis, unaffected by perturbation of the actin cytoskeleton, and independent of ATP. One possibility is that TRPL channels, once released from the rhabdomeres, translocate to the neighboring apical/stalk membrane by simple lateral diffusion within the plasma membrane; adherens junctions would then restrict TRPL channels to the apical membrane. Live imaging studies used to examine diffusion directly were not feasible due to the orientation and geometry of the rhabdomeric and apical membranes involved. We therefore investigated whether perturbations of membrane composition would affect the rate of TRPL translocation. In mammalian cells, membrane fluidity is greatly affected by cholesterol content. In *Drosophila*, the major sterol present is ergosterol [36], [37], which serves a similar role to cholesterol in mammalian cells. Therefore, altering ergosterol content of membranes is expected to affect membrane fluidity. *Drosophila* obtain sterols exclusively from their diet, laboratory-raised flies obtain their ergosterol from the yeast in their food. Yeast, which also have ergosterol as the major sterol present in membranes, in contrast, rely on their own biosynthesis of ergosterol [38], [39]. We previously showed that we could alter ergosterol content of live flies by limiting the ergosterol in their diet [40]. To manipulate the ergosterol intake of flies, we fed flies a specially prepared food made with either wild-type yeast, or a mutant yeast strain with known defects in ergosterol biosynthesis.

**Figure 6:**
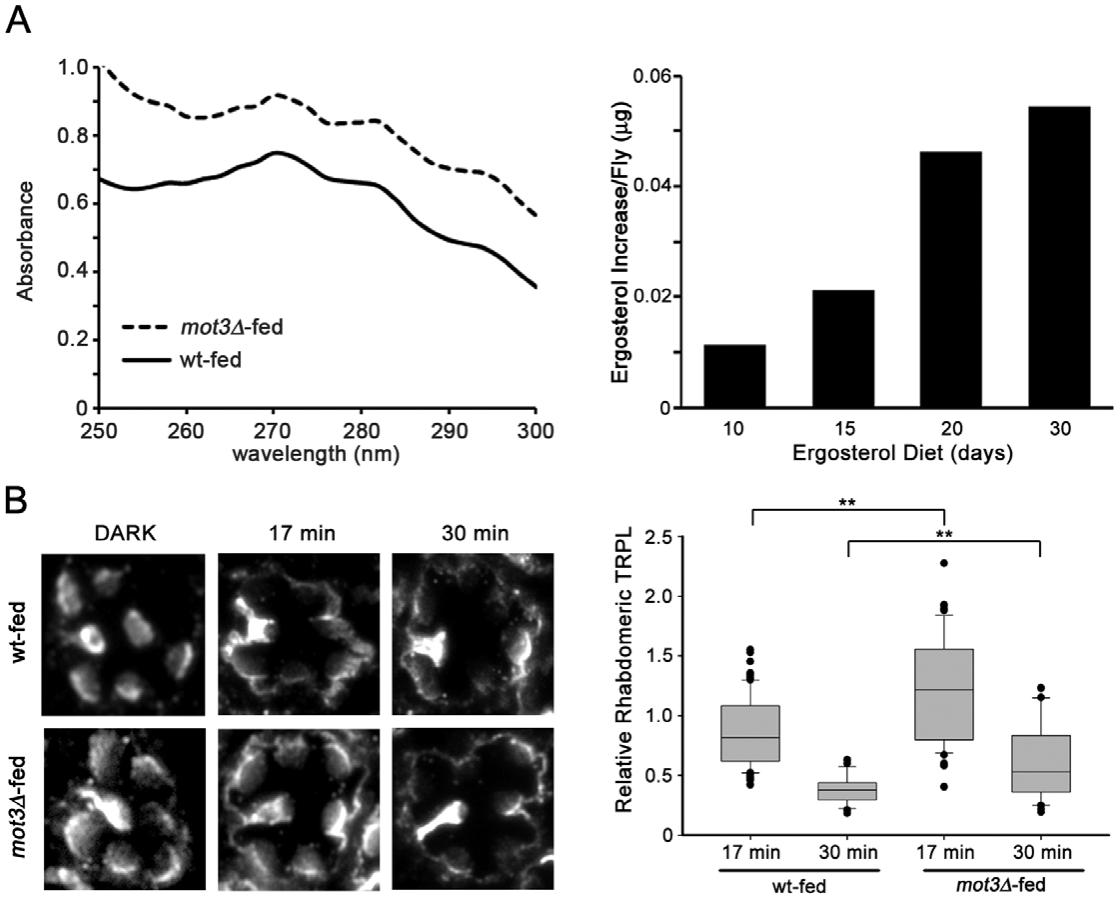
Increased Dietary Ergosterol Slowed the Rate of TRPL Translocation. (A) Wild-type flies were fed a defined medium containing either wild-type or *mot3Δ* mutant yeast for 10, 15, 20 or 30 days in the dark. Sterols were extracted from whole fly membranes and then scanned spectrophotometrically. Left, shown are representative spectral profiles (250–300 nm) of sterols from flies raised for 30 days on food made with wild-type yeast (wt-fed) or *mot3Δ* mutant yeast (*mot3Δ*-fed). This four-peaked profile is characteristic of ergosterol, the major sterol present in *Drosophila*. *mot3Δ*-fed flies consistently displayed an increased absorption spectrum, compared to wt-fed flies. Right, quantification of ergosterol increase in flies fed *mot3Δ*-food, relative to wt-food, for 10, 15, 20, and 30 days. (B) Left, shown are representative retinal sections immunostained for TRPL from wt-fed or *mot3Δ*-fed flies. Dark-raised flies showed rhabdomeric localization of TRPL. With 17 minutes of light-exposure, wt-fed flies displayed a more evident stage-1 TRPL translocation, while flies fed *mot3Δ*-based food displayed a more rhabdomeric-like TRPL localization. After 30 minutes of light-exposure, both wt-fed and *mot3Δ*-fed flies appeared to display stage-1 translocation. Right, Box-and-Whisker-Plots showing the relative rhabdomeric TRPL signal between wt-fed and *mot3Δ*-fed flies when exposed to 17 and 30 min of light. Multiple tissue sections were taken from multiple eyes/flies (across different experiments), as follows. For wild-type, 5 eyes from 4 flies (Dark), 10 eyes from 5 flies (LEX, 17 min), 2 eyes from 2 flies (LEX, 30 min); for *mot3Δ*: 5 eyes from 4 flies (dark), 7 eyes from 5 flies (LEX, 17 min), 3 eyes from 3 flies (LEX, 30 min). ** t-test analysis indicates a significant difference (P<0.01).

For this study, we prepared a more defined diet with yeast from either the *mot3Δ* mutant yeast strain or a control strain with a similar genetic background (wt-yeast). *mot3* encodes a transcriptional repressor of the *ERG2, ERG6*, and *ERG9* genes, which encode enzymes in the biosynthetic pathway of ergosterol, and as expected, *mot3Δ* yeast displays increased ergosterol levels [41]. Wild-type flies were fed a medium containing either wt-yeast or *mot3Δ* mutant yeast for up to 30 days. We compared the ergosterol content of flies raised on these two diets (referred to as wt-food and *mot3Δ*-food) by extracting sterols from whole fly homogenates and subjecting them to ultraviolet spectrophotometric analysis. Ergosterol was identified based on its absorbance profile from 250 to 300 nm similar to previous studies [40], [42]. Spectral profiles were compared for flies fed wt-food and *mot3Δ*-food for 10, 15, 20, and 30 days. Indeed, flies fed *mot3Δ*-food displayed increased absorbance compared to flies fed wt-food at all time points, and this difference increased with longer feeding periods (Figure 6A). By 30 days, flies displayed an increase in ergosterol content of ∼.055 µg per fly.

We then performed immunolocalization studies on flies fed wt-food and *mot3Δ-*food for 25 days to test whether increased ergosterol content resulted in altered rates of stage-1 TRPL translocation. In the dark, TRPL was localized to the rhabdomere in flies fed wt-food or *mot3Δ-*food (Figure 6B). A five minute light-exposure of flies raised on either the wt-food or *mot3Δ-*food, however, failed to induce the robust stage-1 TRPL translocation seen in flies fed our standard laboratory food (data not shown). This difference indicated that the time-course of TRPL translocation was slowed when flies were raised on this minimal diet. When we assayed for rhodopsin-1 (Rh1) levels in these flies by immunoblot analysis, we found that flies raised on either of the minimal diets displayed lower levels of Rh1 than flies raised on our standard fly food (Figure S1). With lower Rh1 levels, it is not surprising that TRPL translocation was slowed. We, therefore, used longer light-exposures to compare TRPL translocation in flies fed wt-food and *mot3Δ-*food. We quantified the relative TRPL channel signal remaining in the rhabdomere after 17 and 30 minutes of light-exposure. We found that TRPL channel translocation at both 17 and 30 minutes was indeed significantly slower in flies fed *mot3Δ-*food, compared to those fed wt-food (Figure 6B). Thus, increasing the sterol content of membranes resulted in slower rates of TRPL translocation, supporting the model that TRPL channels diffuse through the apical membrane during stage-1 translocation.

## Discussion

In this study, we provide further insight into the molecular mechanisms underlying TRPL channel translocation, beginning with the involvement of protein synthesis and then examining factors that could contribute to stage-1 translocation. Given that the second stage of TRPL translocation, as well as its re-localization to the rhabdomere, has been reported to take hours, an open question has been whether these events are indeed due to TRPL protein transport, or degradation and re-synthesis of new TRPL channels. Although protein turnover rates have been examined for TRPL in the blowfly *Calliphora* [7], a direct test of whether protein synthesis is required for the light-dependent redistribution of TRPL channels has not previously been performed. We found that we could feed flies CHX and reliably block new protein synthesis. Our results show that both stages of TRPL channel translocation out of the rhabdomere, even the second stage which takes over 10 hours, do not require protein synthesis, supporting the idea that rhabdomeric TRPL channels are indeed transported out of the rhabdomeres with light-exposure.

We also tested the return of TRPL localization to the rhabdomere with dark-incubation following stage-1 and stage-2 translocation, which require six and ten hours, respectively [8], [9]. We were surprised to find that protein synthesis was required for return from stage-1, but not stage-2. This suggests that the route of re-localization from stage-2 is also likely to be by actual transport of TRPL channels. Furthermore, this pathway from the basolateral membrane to the rhabdomere is distinct from, and does not involve, the path of re-localizing TRPL channels from stage-1. Re-localization from the apical/stalk membrane neighboring the rhabdomere involves protein synthesis. This could imply degradation of these TRPL channels in stage-1 and targeting of newly synthesized TRPL channels to the rhabdomere. Alternatively, transport of TRPL channels from stage-1 to the rhabdomere may require the synthesis of some other protein needed for their mobilization.

In order to use biochemical agents that might be useful in determining mechanisms underlying TRPL translocation, we characterized an *ex vivo* preparation, similar to one previously described [9], amenable to the application of chemical inhibitors. This BHI preparation allows a window of about six hours for retina viability, determined by examining photoreceptor morphology for degeneration, measuring ATP levels in retinas, and noting a light-induced rise in ATP. In this window of viability, we were able to examine mechanisms underlying stage-1 TRPL translocation. To gain insight into whether active or passive transport was likely to be involved, we used inhibitors that would affect ATP levels and the actin cytoskeleton.

When we depleted ATP from retinas, our aim was to see if the loss of ATP affected the light-induced translocation of TRPL channels. Depletion of ATP alone, however, consistently resulted in TRPL channels in the apical/stalk membrane neighboring the rhabdomere, indicating that ATP is required for maintaining channels in the rhabdomere. ATP depletion has previously been shown to activate TRP and TRPL channels [34], [35], and in a further study, these authors suggest that an ATP-dependent process is required to keep the channels closed in the dark [43]. In these studies, the authors propose the following possibilities: 1) ATP binds to destabilize the open state of the channel, similar to ATP-sensitive potassium (KATP) channels [44], 2) constitutive phosphorylation by a protein kinase leads to closure of channels in the dark, 3) an ATP-dependent process is needed to maintain a low concentration of cellular ions, such as Ca^2+^, and 4) ATP depletion results in failure of DAG kinase and/or PI/PIP kinases, leading to accumulation of DAG and/or PIP2 depletion and subsequent channel activation [34], [43]. Since constitutive activation of TRP channels induces TRPL translocation [8], it is likely that activation of TRP/TRPL channels by ATP depletion similarly drives translocation in the dark.

Interestingly, after ATP depletion, TRPL channels are found at the base of the rhabdomeres, as well as throughout the apical/stalk membrane neighboring the rhabdomeres, giving a “ring”-like pattern identical to the pattern seen after light-induced stage-1 translocation. These results suggest that after release from the rhabomeres, TRPL channels translocate to the apical stalk membrane by a passive mechanism, such as lateral diffusion through the membrane. Release of TRPL from the rhabdomeres may involve a Ca^2+^-dependent event triggered with Ca^2+^ influx through TRP channels; anchoring may depend on the N- or C-terminus of TRPL, which have recently been shown to be required for translocation [45]. Lateral diffusion during stage-1 translocation is consistent with the recent finding that stage-1 translocation is independent of Rab5 and RabX4, which mediate vesicular transport of TRPL during stage-2 translocation [46].

Another test for passive versus active transport was the involvement of the cytoskeleton. Actin is the likely component given that the rhabdomeres are made up of microvilli. Disruption of the actin cytoskeleton, however, proved difficult because of the extremely high concentration of actin present. As in previous studies with other rhabdom-based photoreceptors, we found that cytD exposure could be seen to affect the cytoskeleton by the presence of actin asters formed. In these conditions, no change was seen in TRPL translocation. Together with our ATP-depeletion studies, we hypothesized that TRPL channels were translocating by lateral diffusion through the apical membrane. Testing this directly, however, is not easy. Other membrane-protein diffusion studies have measured rates of mobilization at different temperatures, or used GFP-tagged proteins and applied fluorescence recovery after photobleaching (FRAP) techniques. Temperature manipulation, however, would impact not only diffusion of proteins in the membrane, but also enzyme kinetics. Since stage-1 TRPL channel translocation has been shown to require activation of nearly the entire phototransduction cascade [8], multiple enzyme activities would be affected, and results would be difficult to interpret. FRAP-like studies have been impeded by the cellular anatomy, and membrane orientation within ommatidia.

Thus, with a less ideal approach, we aimed to alter membrane composition by increasing the sterol content of membranes, then test for effects on rates of TRPL translocation. We increased sterol content of fly membranes by feeding flies food made from wild-type versus a mutant yeast strain that has increased ergosterol levels. As a result, we found that rates of stage-1 TRPL translocation were indeed slowed. While these results support a model in which TRPL channels, once released from the rhabdomeres, translocate by lateral diffusion throughout the apical membrane, and remain restricted by adherens junctions separating apical and basolateral membranes, this remains to be directly tested with a quantitative evaluation of membrane fluidity and direct measurement of TRPL channel mobility in the membrane. Identification of a light-dependent anchor for TRPL channels in the rhabdomere will also be critical for validating and understanding the proposed diffusion-based translocation.

## Materials and Methods

### Fly Stocks

All fly stocks were raised in the dark at 25°C and were fed standard fly food consisting of cornmeal, yeast, molasses and agar, unless otherwise noted. *cn bw* and *w^1118^* lines were used as wild-type. Transgenic *inaD^1^* null line expressing *inaD* under the control of the heat-shock promoter were generated previously, described in [47]. The *shibire^ts1^* mutant line was obtained from the Bloomington *Drosophila* Stock Center.

### Light-Exposure of Flies

For light-exposure, dark-raised flies less than one week old were placed in vials containing standard fly food, unless otherwise noted, and covered with clear plastic wrap. Holes were punctured in plastic wrap using forceps to provide adequate ventilation. Flies were placed 15 cm from a white light source (Lambda LS 175W Xenon-arc lamp with 400–700 nm bandpass filter, Sutter Instruments, Novato, CA, or equivalent) for given times. Light intensity was measured by an EXTECH 403125 digital light-meter. All experiments were conducted at room temperature. Light intensities used for inducing stage-1 and stage-2 TRPL translocation were ∼2297 and ∼244 lux, respectively, unless otherwise noted. Light intensities are within a physiological range; as a reference, room light is ∼500–1000 lux, a sunny day in the shade is ∼4500 lux, and a sunny day in direct sun is ∼54×10^3^ lux.

### Cryosectioning and Immunostaining Retinal Sections

After illumination, fly heads were skewered onto stainless steel minutien pins (Fine Science Tools, Foster City, CA) and fixed in 3% paraformaldehyde and 5 mM ethylenediaminetetraacetic acid (EDTA) in phosphate buffered saline (PBS), washed with PBS, and infiltrated with 2.3 M sucrose in PBS overnight at 4°C. Dark-raised flies were fixed under a dim red light before sectioning. Heads were bisected, eyes oriented to face upward on an ultramicrotomy pin (Ted Pella, Redding, CA), and frozen in liquid nitrogen. 1–1.5 µm thick sections were cut from retinas using a Leica Ultracut with EM FCS cryo unit at −81°C (Leica Microscopy and Scientific Instruments Group, Heerbrugg, Switzerland). Sections were blocked, then incubated with primary antibody overnight at 4°C. Slides were washed with 0.1% sapponin in PBS. An FITC or rhodamine-conjugated secondary antibody (Jackson ImmunoResearch, West Grove, PA) diluted 1∶200 in blocking solution was used for 1 hour at room temperature in the dark. Slides were washed again and mounted with 90% glycerol, 10% 1 M Tris (pH 8.5) and 0.1% *p*-phenylenediamine (Sigma Aldrich, St. Louis, MO). Images were taken with an Olympus MagnaFire 2.0 camera S99806, and processed in Adobe Photoshop for illustration.

### Quantification of TRPL Signal in Retinal Sections

Rhabdomeric TRPL signal was measured and quantitated from images of 1 µm thick retinal sections immunostained for TRPL. Using ImageJ, TRPL fluorescent signal within each rhabdomere was quantified by measuring the total signal within a circle of fixed diameter (approximate area a single rhabdomere) placed on each rhabdomere using a corresponding phase-contrast image. Five ommatidia were selected from each tissue section, and signal from three rhabdomeres in each ommatidium were measured. Three similar measurements were taken outside of the ommatidia to calculate an average background value for each section; this background value was subtracted from every TRPL signal measurement. For statistical purposes, the average TRPL signal from any given ommatidium was considered an independent measurement. TRPL signal from light-exposed samples were normalized to the TRPL signal intensity from dark-raised flies in the same experiment. A three-standard deviation limit was used to remove outliers. Data sets from 17 min and 30 min light-exposed flies passed a normal distribution test, enabling us to apply the student's t-test to identify statistical differences.

### Cycloheximide Feeding Protocol

Five day old wild-type flies were starved, given only water on a moistened cotton ball for 24 hours. The control group was subsequently fed 3% sucrose while the experimental group was fed 35 mM cycloheximide (CHX) (synonym: actidione, Sigma Aldrich, St. Louis, MO) in 3% sucrose. Both solutions were dyed with green food coloring and mixed in equal amounts with instant fly food (Carolina, Burlington, NC). Flies were fed +/− CHX for at least 30 minutes in either dark or light conditions, then subsequently dark incubated or light-exposed. Flies remained on the +/− CHX food throughout the duration of the dark incubation or light exposure. Only dark-green flies were selected for analysis.

### Bisected Head Illumination (BHI) Preparation

Fly heads were removed and bisected under dim red lighting. Eyes were incubated in a 24-well plate containing 650–750 µl per well of a bath solution (in mM: 120 NaCl, 5 KCl, 10 TES Buffer (N-Tris (hydroxymethyl)-methyl-2-amino-ethanesulphonic acid, pH 7.15), 4 MgSO4, 1.5 CaCl2, supplemented with 5 sucrose, and 5 trehalose). The eyes floated in mixed orientations in the bath. A maximum of 10 eyes per well were incubated in the bath solution. For light exposure, the 24-well plate was placed on a nutating mixer and rocked 28 cm from the white light source (Lambda LS 175W Xenon-arc lamp, 400–700 nm bandpass filter, Sutter Instruments, Novato, CA, or equivalent) for the indicated times. Light intensity was measured by an EXTECH 403125 digital light-meter. Plates containing dark-raised samples were wrapped in red plastic wrap and aluminum foil and simultaneously rocked on the nutator. After illumination, the bath solution was gently removed from the well with a pipette. The eyes remained in the same well and were fixed with 3% paraformaldehyde and 5 mM EDTA in PBS for 30–60 minutes, rinsed with PBS, infiltrated with 2.3 M sucrose in PBS overnight at 4°C, then cryosectioned and immunostained as described above.

### ATP Depletion and Quantification

Dark-raised wild-type eyes were incubated in bath solution without sucrose and trehalose, supplemented with 10 mM potassium cyanide (KCN) and 5 mM 2-deoxy-D-glucose for 60 minutes, in the dark at room temperature and placed on the nutating mixer. Eyes were fixed, rinsed and infiltrated with sucrose. To measure the ATP concentration in the eyes, 6 eyes were homogenized in 50 µl of deionized water and the ATP content of the homogenized sample was determined using an ATP Determination Kit (Molecular Probes, Eugene, OR) according to the provided protocol.

### Mutant Yeast Fly Food

For specialized yeast fly foods, the *mot3*Δ (FY2071 [41]) mutant strain and an appropriate wild-type background strain FY86 (similar to FY2066 [41]) were used. YPD medium (2% Glucose, 1% Yeast extract, 2% Peptone) was autoclaved for 15 minutes to culture the mutant strains. Single mutant colonies for all strains were selected from plates and added to 50 ml of YPD medium and cultured on an orbital shake at 200 rpm, overnight at 30°C. Cultures were centrifuged at 1000×g for 10 minutes. Yeast pellets were washed once with 100 ml of water, and centrifuged again at 1000×g for 10 minutes. To cook mutant yeast fly food, 1 g agar, 5 g glucose, and 50 ml water were heated together to 80°C. Six grams of mutant yeast (pellets) was subsequently added and mixed until the mixture was homogeneous. After the food cooled to 70°C, 500 µl of methyl-4-hydroxybenzoate (stock solution of 106.6 g/1 L ethanol; Sigma Aldrich, St. Louis, MO) was added to the food to prevent mold growth.

### Sterol Extraction

For each sample, 15–50 flies were homogenized in 160 µl of water. The homogenate was centrifuged at 6000 rpm for 4 minutes at room temperature in a microcentrifuge to remove chitin and the supernatant was collected. The pellet was resuspended in 80 µl of water and spun again. The supernatants were collected; 20 µl were used to perform a Bradford Protein Assay to quantitate total protein concentration. Volumes of supernatant samples were normalized for protein concentration and transferred to glass tubes. 3 ml of 25% alcoholic potassium hydroxide solution was added to each sample, followed by 1 minute of vortexing. Sample was incubated at 80°C for 1 hour, and allowed to cool to room temperature. Sterols were extracted by adding 1 ml of water and 3 ml of *n*-heptane, followed by vigorous mixing/vortexing. The solution was allowed 5 minutes to settle after which a clear interface between layers was visible. 2.7 mls of the upper *n-*heptane layer was carefully removed and transferred to a new glass tube. Samples were dried under a steady stream of nitrogen gas to slow down the quick oxidation of ergosterol while warmed at 48°C (∼20–30 minutes). Dried samples were resuspended in 120 µl of 100% ice-cold ethanol. 100 µl of each sample was immediately analyzed for absorbance between 250 and 300 nm. Expected ergosterol peaks are at 260 nm, 270 nm, 282 nm, and 294 nm. The extinction coefficient for ergosterol in alcohol at λ_282_ is 10,500 M^−1^ cm^−1^, which was used for quantifying ergosterol differences between samples.

## Supporting Information

Figure S1 **Rhodopsin-1 Levels are Lower in Flies Fed Defined Diet.** Representative immunoblot of fly head homogenates from wild-type flies fed either a defined diet containing wild-type yeast (wt-fed) or *mot3Δ* mutant yeast (*mot3Δ*-fed), or standard laboratory fly food (std-fed). Immunoblots were probed using antibodies against rhodopsin-1 (Rh1), or syntaxin (syn) as a loading control (3 heads/lane).
(DOCX)
