# Supplementary material for: Mechanisms Underlying Stage-1 TRPL Channel Translocation in Drosophila Photoreceptors
Source: PLoS One. 2012 Feb 20;7(2):e31622. doi: 10.1371/journal.pone.0031622 (PMC3282777; doi:10.1371/journal.pone.0031622)
Supplement: Figure S1 — Rhodopsin-1 Levels are Lower in Flies Fed Defined Diet. Representative immunoblot of fly head homogenates from wild-type flies fed either a defined diet containing wild-type yeast (wt-fed) or mot3Δ mutant yeast (mot3Δ-fed), or standard laboratory fly food (std-fed). Immunoblots were probed using antibodies against rhodopsin-1 (Rh1), or syntaxin (syn) as a loading control (3 heads/lane). (DOCX) [file pone.0031622.s001.docx]

Figure S1


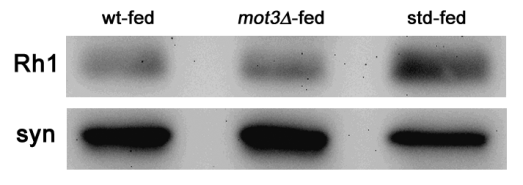


**Figure S1.** Rhodopsin-1 Levels are Lower in Flies Fed Defined Diet.

Representative immunoblot of fly head homogenates from wild-type flies fed either a defined diet containing wild-type yeast (wt-fed) or *mot3∆* mutant yeast (*mot3∆*-fed), or standard laboratory fly food (std-fed). Immunoblots were probed using antibodies against rhodopsin-1 (Rh1), or syntaxin (syn) as a loading control (3 heads/lane).
